# Supplementary material for: Mapping robust multiscale communities in chromosome contact networks
Source: Sci Rep. 2023 Aug 10;13:12979. doi: 10.1038/s41598-023-39522-7 (PMC10415398; doi:10.1038/s41598-023-39522-7)
Supplement: Supplementary file 1 — Supplementary Information. [file 41598_2023_39522_MOESM1_ESM.pdf]

Supplementary Information for "Mapping robust multiscale communities in chromosome contact networks"

Anton Holmgren, Dolores Bernenko, and Ludvig Lizana†

Integrated Science Lab,
Department of Physics,
Umeå University, Umeå,
Sweden
†*Corresponding author: ludvig.lizana@umu.se*

(Dated: December 29, 2022)

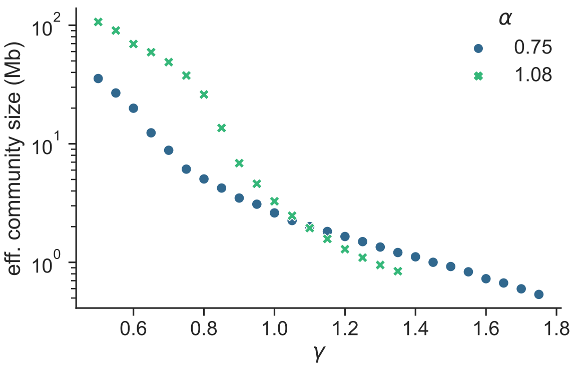

FIG. S1 Effective community size for different scale parameters γ and decay parameters α for chromosome 10.

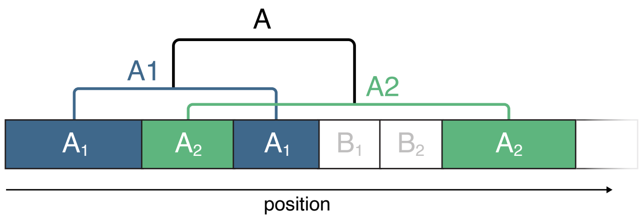

FIG. S2 Schematic structural scales derived from A1/A2/B1/B2/B3 sub-compartment data.? The smallest organisational scale is $A_{1,2}/B_{1,2,3}$-segments, a contiguous DNA stretch that fully belongs to a sub-compartment. The next scale is A1/.../B3 sub-compartments – collections of DNA segments. The largest structural scale is the A/B compartments.

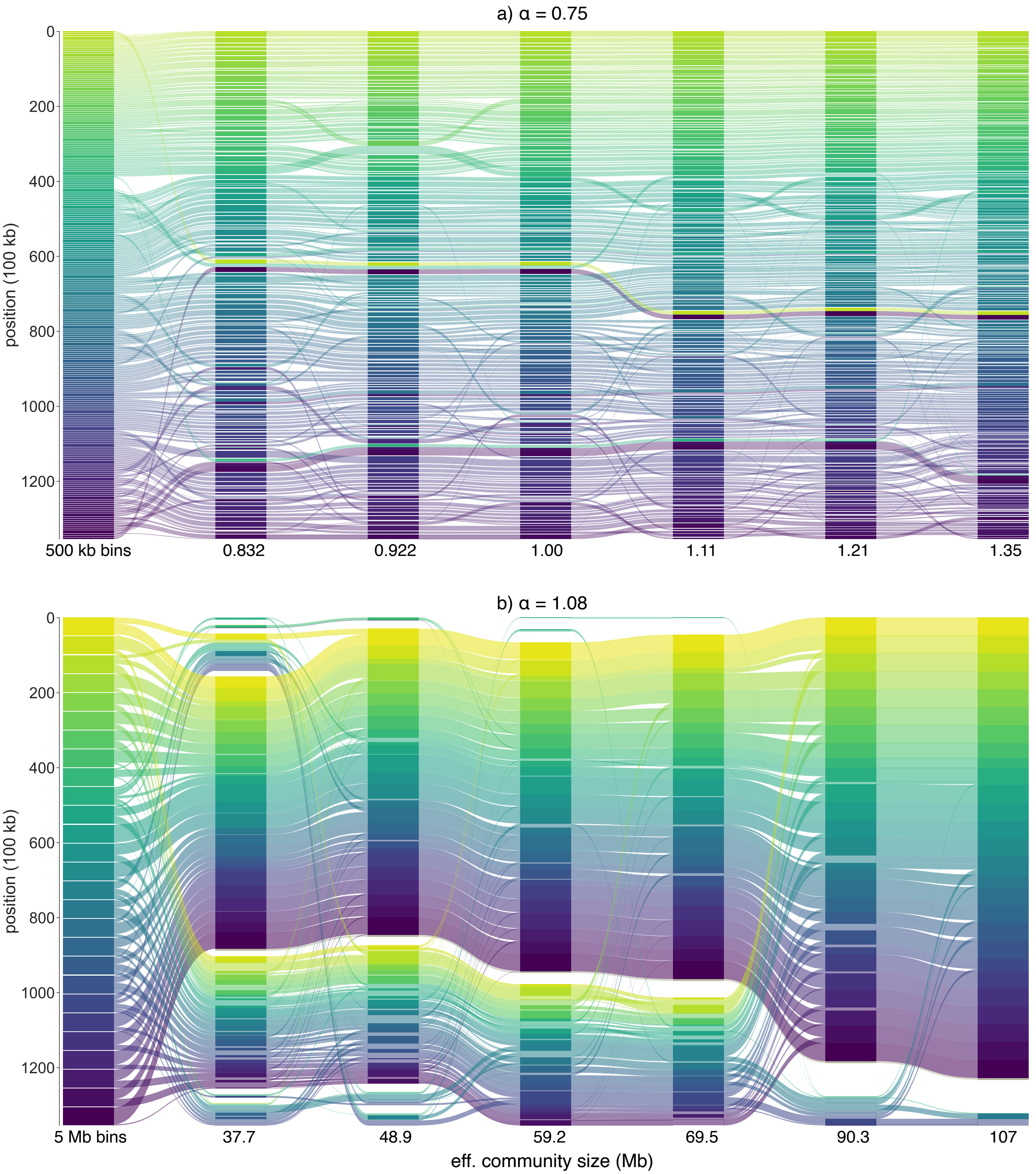

FIG. S3 Alluvial diagram of core communities of chromosome 10 for $\alpha = 0.75$ and $\alpha = 1.08$ at different scales. The left-most column represents linear bins coloured by position. The remaining columns represent community structure at different scales, vertically ordered by their average position and coloured by the positions of their contained segments. Transparent segments are not in the core.
